# Characterization of Hydrogel Beads for the Gradual Release of *Origanum vulgare* L. Essential Oil and Evaluation of Their Antifungal Activity Against *Candida albicans*

**DOI:** 10.3390/microorganisms13092065

**Published:** 2025-09-05

**Authors:** Victoria Concha, Mario Díaz-Dosque, Luisa Fernanda Duarte, José A. Jara, Alfredo Molina-Berríos

**Affiliations:** 1 Institute for Research in Dental Sciences, Faculty of Dentistry, University of Chile, Olivos 943, Independencia, Santiago 8380544, Chile; victoria.concha@ug.uchile.cl (V.C.); mrdiaz@uchile.cl (M.D.-D.); jsandovalj@uchile.cl (J.A.J.); 2 Centro de Medicina Regenerativa, Facultad de Medicina, Clínica Alemana, Universidad del Desarrollo, Santiago 7610658, Chile; lduarte@udd.cl; 3 Millenium Institute on Immunology and Immunotherapy, Santiago, Chile

**Keywords:** oregano essential oil, *Candida albicans*, hydrogel beads, controlled release, alginate

## Abstract

*Candida albicans* infections are associated with high morbidity and mortality worldwide. Current antifungal therapies are limited by adverse effects and the emergence of resistant strains, which compromise long-term efficacy. Previous studies have shown that *Origanum vulgare* L. essential oil (OvEO) possesses strong antifungal activity; however, its volatility and physicochemical instability hinder clinical application. The aim of this study was to encapsulate OvEO in a hydrogel and evaluate its release kinetics, chemical composition, structural properties, and antifungal activity. We assessed its release kinetics, chemical composition, structural characteristics (FTIR; SEM), and antifungal activity against *C. albicans*. OvEO was successfully encapsulated into hydrogel beads, enabling a gradual release profile, with in vitro release of phenolic compounds reaching 100% at 48 min. SEM revealed an irregular surface with small pores and crystalline aggregates distributed across the bead surface. OvEO-loaded hydrogel beads inhibited *C. albicans* growth with an IC_50_ of 0.15 ± 0.05 mg/L for strain 90029 and 0.2 ± 0.06 mg/L for strain 10231. At these concentrations, adhesion to abiotic surfaces was reduced by 60–80%. These findings support the potential of OvEO-loaded hydrogel beads as an alternative approach for the treatment of fungal infections, offering a complementary strategy to current antifungal agents.

## 1. Introduction

Yeasts of the genus *Candida* spp. are opportunistic human pathogens that typically exist as harmless commensals within the human microbiota, colonizing the mouth, throat, gastrointestinal tract, vagina, and skin. However, under conditions of microbiota disruption or immune system compromise, these organisms can transition to a pathogenic state, leading to localized or systemic infections [1,2]. Recent global reports estimate that *Candida* spp. are responsible for approximately 400,000 new cases of potentially life-threatening candidiasis each year, with mortality rates ranging from 40% to 60%, even with antifungal treatment [3,4]

Non-*albicans Candida* species, such as *C. glabrata*, *C. tropicalis*, *C. parapsilosis*, and *C. krusei*, are increasingly recognized as significant opportunistic pathogens. They pose a growing challenge in clinical practice due to their rising prevalence, intrinsic or acquired resistance to antifungal agents, and their association with severe systemic infections, particularly in immunocompromised patients. Nevertheless, among *Candida* species, *C. albicans* is the most clinically significant. In fact, it was recently classified as a critical priority pathogen in the World Health Organization’s 2022 fungal priority pathogens list, primarily due to its public health burden, global incidence, wide geographic distribution, and increasing resistance to antifungal agents [5]. Additionally, *C. albicans* is considered the most virulent species and the predominant yeast responsible for human infections, accounting for approximately 50% of all cases. It is implicated in a wide range of clinical manifestations, including invasive candidiasis, vulvovaginal candidiasis, esophageal candidiasis, cutaneous candidiasis, and oral candidiasis [6,7].

Therapeutic management of candidiasis, in any of its clinical forms, should be tailored to the severity of the condition and the degree of immunosuppression of the patient. Topical antifungal therapy is considered the first-line treatment for uncomplicated cases. Systemic therapy is reserved for patients who are refractory or intolerant to topical treatment, as well as for those at higher risk of developing systemic infections [8].

There is currently a growing disparity between the increasing prevalence of fungal infections and the development of new antifungal agents. This gap is reflected in the scarcity of new effective and safe antifungal drugs introduced since the early 2000s [9,10]. In fact, the current antifungal treatment arsenal remains quite limited, as only three major drug classes are available for the treatment of *C. albicans* infections: polyenes, azoles, and echinocandins. Besides the restricted range of therapeutic options, these agents exhibit various limitations and pharmacological drawbacks that compromise treatment efficacy and hinder effective patient management [11]. Therefore, the development of reliable and alternative therapeutic agents to conventional antifungal compounds has become an urgent priority for the effective treatment of *C. albicans* infections [12,13] In this context, natural products, particularly essential oils (EOs) have emerged as promising sources of novel bioactive compounds, with antifungal activity comparable to, or even surpassing, that of currently available antifungal drugs [14,15,16,17].

Essential oils (EOs) are hydrophobic fluids composed of complex, low-molecular-weight (<500 Da) volatile molecules, synthesized by plants as secondary metabolites. They fulfill multiple roles, including acting as part of the plant’s immune system, facilitating interactions with microorganisms, attracting pollinators, and participating in protective responses against environmental stressors [18]. Their chemical composition is influenced by a range of factors associated with the producing plant, including geographical location, ecotype or variety, biological and physicochemical properties of the soil, nutrient availability, fertilizer use, and seasonal variations [19]. Each EO can contain between 20 and 100 secondary metabolites of diverse chemical origin, and the presence or combination of these compounds determines the biological and pharmacological activities attributed to them [20].

EOs derived from plants of the *Lamiaceae* family, such as *Origanum vulgare* L., are particularly noteworthy due to their high content of terpenes with well-documented antifungal properties. These compounds have demonstrated the ability to inhibit both growth and biofilm formation in various *Candida* spp., as well as the suppression of morphogenesis [15,16].

Evidence suggests that EOs could be considered a viable therapeutic alternative; however, due to their plant-based origin, they possess certain characteristics such as being hydrophobic, volatile, and environmentally labile, as they are prone to oxidation and degradation. Moreover, at high concentrations, they may exhibit toxicity [12,20]. Therefore, it is necessary to develop a pharmaceutical formulation that ensures a non-immediate or gradual release of EO components at low concentrations to minimize toxicity, compensate for their susceptibility to degradation and oxidation, and ultimately achieve effective or efficient bioavailability against pathogens [21].

A variety of pharmaceutical formulations are currently available as potential alternatives for encapsulating EOs, thereby protecting them from environmental factors, preserving their properties, improving their stability, preventing degradation, and enabling controlled release. Examples include emulsions, nanoparticles, liposomes, nanofibers, and hydrogels, among others. The method of choice depends on the component to be encapsulated, the physicochemical properties of the coating material, cost considerations, and the intended application [22,23]. Considering this, hydrogels exhibit several characteristics that make them particularly suitable for evaluation as delivery vehicles for EOs.

Hydrogels are three-dimensional structures composed of a network of natural or synthetic polymers, stabilized by crosslinking agents. They are insoluble, highly permeable, biocompatible, biodegradable, hydrophilic, and capable of absorbing large amounts of water, which allows them to promote a moist environment [23,24]. Specifically, alginate-based hydrogels have proven particularly attractive for numerous biomedical applications, including wound healing, the delivery of bioactive agents such as small drugs and proteins, tissue engineering, and cell transplantation. This is due to their structural similarity to the extracellular matrices of living tissues, their ability to be administered orally or injected in a minimally invasive manner, and their capacity to be engineered to perform various critical functions [25]. Regarding the use of alginate hydrogels for the protection and controlled release of bioactive molecules and drugs, numerous studies have demonstrated their effectiveness. For example, the encapsulation of drugs such as doxorubicin, methotrexate, and flurbiprofen in alginate hydrogels has facilitated their controlled release and improved their efficiency. Studies have also reported the successful controlled and localized release of antineoplastic agents using partially oxidized alginate gels [25,26]. Finally, recent publications indicate that this type of pharmaceutical formulation can also serve as a vehicle for the encapsulation and release of EOs, exhibiting strong antibacterial and anti-inflammatory activity [23,27]. To the best of our knowledge, no investigations have addressed the use of alginate hydrogel beads containing *O. vulgare* L. essential oil as an antifungal/antibiofilm approach against *C. albicans*, indicating a notable gap in the existing literature. Therefore, the aim of this study was to encapsulate OvEO into alginate hydrogel beads and to assess their release characteristics and in vitro antifungal performance against planktonic and sessile cultures of *C. albicans* strains.

## 2. Materials and Methods

### 2.1. Plant Material and Extraction of Essential Oil

The leaves of *O. vulgare* L. were collected during spring flowering in Chicauma, Lampa, Metropolitan Region, Chile (33°14′23″ S, 70°54′27″ W), and identified as previously reported (specimen number CONC 191040). The essential oil was obtained by hydrodistillation using a Clevenger-type apparatus for 4 h. For this process, 200 g of dried *Origanum vulgare* L. leaves were ground into powder using a mechanical grinder and immersed in 1 L of distilled water in a round-bottom flask. The resulting essential oil was aliquoted into 1.5 mL amber tubes and stored at −20 °C (extraction yield was 0.77%) [15].

### 2.2. Preparation of Origanum vulgare L. Essential Oil Emulsion

O/W emulsions were prepared by ultrasonication. OvEO was incorporated into a 2% (*w*/*v*) sodium alginate hydrogel and homogenized at 15,500 rpm for 5 min using a Tissue Master 125 (Omni, Kennesaw, GA, USA). Six emulsions, each containing different essential oil concentrations, were subsequently formulated as follows (Table 1).

### 2.3. Ionotropic Gelation for Bead Formation

The alginate beads for the encapsulation process were obtained as previously described [28]. A hardening solution was prepared by dissolving 3 g of CaCl_2_ in 100 mL of distilled water. Hydrogel beads were generated by adding 20 µL of each OvEO emulsion to the hardening solution using a 1 mL syringe at a distance of 3 cm between the dripping tip and the liquid surface. Once the beads were formed, they were immediately removed from the solution using a Pasteur pipette (Corning, New York, NY, USA). The average time required to remove each bead was approximately 5 s.

### 2.4. Scanning Electron Microscopy (SEM)

The OvEO-loaded hydrogel beads were analyzed using scanning electron microscopy (SEM). The samples were lyophilized and fixed in 50% alcohol, followed by dehydration in a graded alcohol series for 5 min each: 70%, 95%, 100% I, and 100% II. Subsequently, they were placed in a critical point dryer, Autosamdri-815, Series A Overview, for approximately 30 min. Once dried, they were mounted on aluminum stubs and then coated with carbon using a Desk V sputter coater (Denton Vacuum, Morestown, NJ, USA). Finally, the samples were visualized using a scanning electron microscope (model JSM IT300LV, JEOL, Tokyo, Japan) operated at a voltage of 15 kV, and their elemental composition was determined by energy-dispersive X-ray spectroscopy (EDX) coupled with SEM.

### 2.5. Fourier Transform Infrared Spectroscopy (FTIR)

In order to verify alginate crosslinking, detect OvEO incorporation, and assess potential matrix–oil interactions, FTIR was performed on empty and loaded beads. Samples were prepared by lyophilizing the unloaded and OvEO-loaded hydrogel beads. FTIR-ATR (Agilent Technologies, Model Cary 630, Santa Clara, CA, USA) equipped with the MicroLab Expert software (v 5.20) was used. The samples were placed in direct contact with an attenuated total reflectance (ATR) crystal, characterized by a high refractive index. As the infrared beam passes through the crystal, it reflects at least once on the internal surface in contact with the sample, producing a spectrum that represents molecular absorption and transmission. Data were collected and analyzed in a range of 400–4000 cm^−1^, with 20 scans recorded at a <2 cm^−1^ resolution.

### 2.6. C. albicans Strains and Growth Conditions

Two reference strains were used to evaluate the effect of OvEO-loaded hydrogel beads. The fluconazole-susceptible *C. albicans* 90029 and the fluconazole-resistant *C. albicans* 10231 strains were both obtained from ATCC and maintained on Sabouraud chloramphenicol agar plates (Biokar, Beauvais, France) at 4 °C until use. For each experiment, preinocula were prepared from a single colony (1–2 mm diameter) in RPMI-1640 medium (Sigma-Aldrich, St. Louis, MO, USA) and maintained in an incubator (Biobase, BJPX-H160, Jinan, China) under planktonic growth conditions (28 °C) for 16–18 h to suppress hyphal induction and enable accurate hemocytometer counts. Standardized inocula were then used for all assays conducted at 37 °C in RPMI-1640.

### 2.7. Inhibition of C. albicans Growth

Inhibition of *C. albicans* growth was evaluated essentially as described previously [15]. First, a standardized suspension (0.5 × 10^5^ UFC/mL) in RPMI-1640 media (Sigma-Aldrich) was prepared from a preinoculum by manually counting using a Neubauer chamber (Blaubrand, Germany). Then, the assay was performed by inoculating each well of a 96-well flat-bottom polystyrene microplate (Nest, Wuxi, China) with 100 µL of the suspension and 100 µL of RPMI-1640 media. Subsequently, one hydrogel bead loaded with different concentrations of OvEO (0–3%) was added to each well, and the plate was incubated at 37 °C without shaking for 24 h (Nuaire NU-5800, Plymouth, MN, USA). After incubation, hydrogel beads were carefully removed using a tuberculin syringe needle. Finally, growth inhibition was determined by measuring optical density at 450 nm after shaking the plate for 5 s at normal intensity using a microplate reader (Infinite F50, Tecan, Zurich, Switzerland). Negative controls contained hydrogel beads without OvEO loading. Fluconazole (0.1–64 mg/L) was used as a positive control. The concentrations of OvEO loaded in hydrogel beads capable of inhibiting the growth of the tested strains by 50% (IC_50_) were determined by constructing dose–response curves using GraphPad Prism 8.0 and subsequently used as reference values in the following assays.

### 2.8. Adhesion Assay

The assay was performed essentially as previously reported with minor modifications [15]. First, each well of a 96-well polystyrene microplate (Falcon, Corning, USA) was inoculated with 100 µL of a standardized suspension of *C. albicans* (1 × 10^6^ CFU/mL), in the presence or absence of OvEO-loaded hydrogel beads at three different loading concentrations (0.5 × IC_50_, IC_50_, and 2 × IC_50_), as well as fluconazole at its respective IC_50_. The plates were incubated for 4 h (initial adherence) at 37 °C (Nuaire NU-5800) without shaking in RPMI-1640 medium supplemented with fetal bovine serum (FBS) (10% *v*/*v*) to promote yeast filamentation and adhesion. After incubation, hydrogel beads were carefully removed using a tuberculin syringe needle, non-adherent cells were discarded, and wells were washed three times with 100 µL of phosphate-buffered saline 1X (PBS, Merck, Darmstadt, Germany). Subsequently, 100 µL of 0.1% crystal violet (CV) solution (Amresco LLC, Solon, OH, USA) was added to each well, and the mixture was incubated for 5 min at room temperature to allow for staining of the adherent cells. The CV solution containing non-adhered cells was discarded, and the adhered cells were carefully washed 4 to 5 times with PBS. To visually assess the effect of the treatments on the adhesion process, the wells were photographed using a digital camera (Optika C-B5, Ponteranica, Italy) mounted on an inverted optical microscope (Lieder MI-530, Ludwigsburg, Germany) and analyzed with the Optika ProView software (v 4.11). Afterward, 100 µL of acetic acid (10% *v*/*v*) (Sigma-Aldrich) was added to each well and incubated for 15 min at room temperature to solubilize the dye. Finally, the supernatant was read at 570 nm after shaking the plate for 5 s at low intensity using a microplate reader (Infinite F50, Tecan). The results were expressed as the mean percentage of adhesion relative to the untreated control group.

### 2.9. Essential Oil Release Profile from the Hydrogel Beads

EO release from the hydrogel beads was quantified by spectrophotometric analysis at 272 nm using a UV–Vis spectrophotometer (Genesys 150, Thermo Fisher, Waltham, MA, USA). Due to the complex composition of the OvEO, its main water-soluble component, thymol, was selected as a marker for quantification [15]. A standard calibration curve for thymol was constructed prior to the analysis (0.006–0.05 mg/mL). Accordingly, the results of EO release from the beads were expressed as thymol equivalents by using the equation obtained from the calibration curve.

For the OvEO release profile determination, 18 hydrogel beads were prepared with the IC_50_ obtained for each strain and added to a beaker with 2 mL of RPMI-1640 medium (Sigma-Aldrich). Subsequently, aliquots of 2000 µL were taken and transferred to a quartz cuvette for absorbance measurement at 272 nm every 3 min. Then, the aliquots were returned to the beaker. The absorbance values obtained from the measurements of the beads loaded with OvEO were substituted into the thymol calibration curve equation established initially to determine the thymol concentration in mg/mL. To determine the percentage of thymol release, the concentration recorded after mechanical disintegration of all the hydrogel beads was considered a 100% release. Based on this, the percentage was calculated by substituting the maximum thymol concentration value recorded for the non-disintegrated hydrogel beads into the following formula:

$$\frac{\left[Thymol\;mg/mL\right]\;maximum\;release\;from\;hydrogel\;beads\times 100}{\left[Thymol\;mg/mL\right]\;desintegrated\;hydrogel\;beads}=\%\;Thymol\;release$$

The maximum thymol concentration value obtained from the disintegration of the 18 hydrogel beads was divided by 18 to obtain the thymol concentration in a single hydrogel bead, and knowing the percentage of thymol in the OvEO used (14.2%) according to its previous characterization [15], the OvEO concentration contained in a single hydrogel bead was calculated using the following equation:

$$[OvEO\;1\;hydrogel\;bead\;µg/mL]=\frac{\left[Thymol\;1\;hydrogel\;bead\;µg/mL\right]\times 100}{14.2}$$

### 2.10. Statistical Analysis

All assays were performed in quadruplicate and independently repeated at least three times (n = 3). Results were expressed as mean ± SD and analyzed using one-way ANOVA (GraphPad Prism 8.0). Results were considered statistically significant when *p*-values were <0.05.

For the quantification of OvEO release from the hydrogel beads, a linear standard curve of thymol was first constructed, and the equation of the line, y = mx + c, was calculated to interpolate the absorbance data corresponding to OvEO release from the hydrogel beads (Origin Pro 2018).

## 3. Results

### 3.1. Morphology and Analysis of Hydrogel Beads Loaded with OvEO

**Figure 1:**
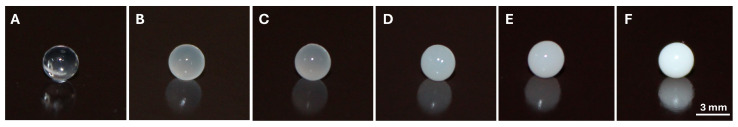
Photographic record of hydrogel beads at different concentrations. (**A**) Hydrogel bead without OvEO. (**B**) Hydrogel bead with 0.01% OvEO. (**C**) Hydrogel bead with 0.2% OvEO. (**D**) Hydrogel bead with 0.5% OvEO. (**E**) Hydrogel bead with 1% OvEO. (**F**) Hydrogel bead with 3% OvEO.

Hydrogel beads were successfully formulated using sodium alginate and calcium chloride as crosslinking agents, incorporating OvEO at various concentrations. The resulting beads displayed a uniform spherical shape and an average diameter of approximately 3 mm, with no visible signs of phase separation or oil leakage (Figure 1). Additionally, as the amount of OvEO incorporated into the alginate hydrogel increased, the resulting beads became less transparent and exhibited a more whitish appearance (Figure 1A–F). This visual change may be attributed to the dispersion of oil droplets within the hydrogel matrix, which increases light scattering and reduces translucency, indicating higher oil loading within the network

**Figure 2:**
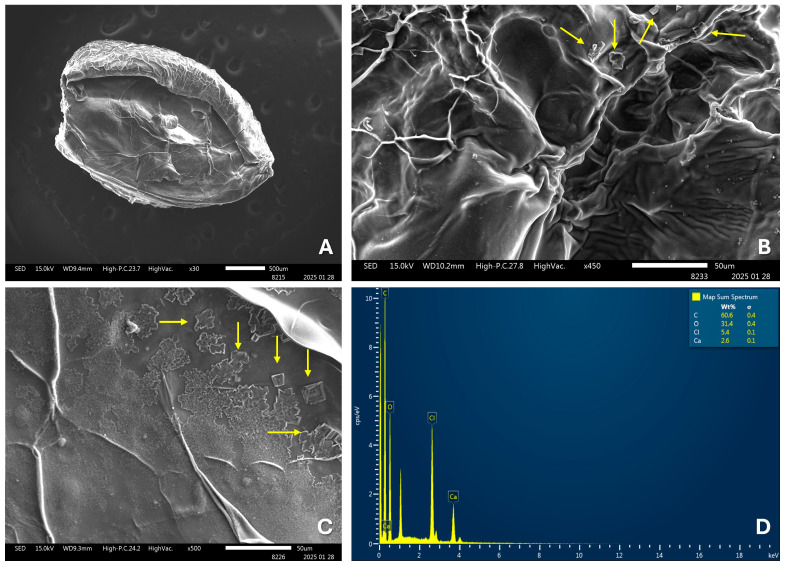
SEM images of hydrogel beads at magnifications of 30× (**A**), 450× (**B**), and 500× (**C**). Arrows highlight CaCl_2_ crystal deposits distributed in the structure in (**B**,**C**). Energy-dispersive X-ray spectroscopy (EDX) of OvEO-loaded hydrogel beads (**D**).

SEM analysis of the OvEO-loaded hydrogel beads revealed an irregular surface morphology with the presence of creases and porosity, attributed to matrix shrinkage induced by lyophilization and the sublimation of water within the polymeric matrix (Figure 2A–C). Additionally, the presence of crystal deposits distributed throughout the structure was observed and analyzed by energy dispersive X-ray spectroscopy (EDX) coupled with SEM (Figure 2D). These structures were identified as residual CaCl_2_ crystal deposits, attributable to the use of the calcium chloride gelling solution employed in the formation of the hydrogel beads.

**Figure 3:**
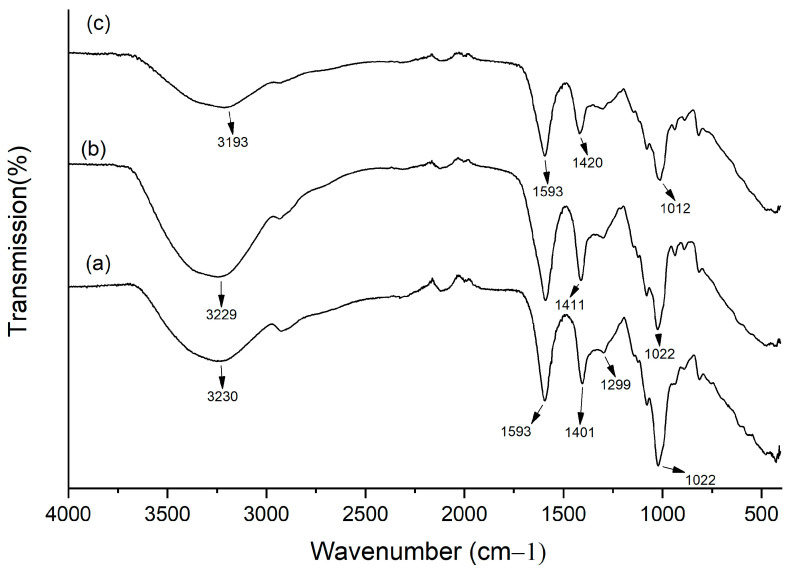
FTIR spectra of lyophilized (**a**) alginate; (**b**) alginate–calcium; and (**c**) alginate–calcium-OvEO. Arrows indicate characteristic signals as described in the text.

To evaluate if the components of the system could interact, we performed FTIR analysis on alginate alone and in the presence of calcium and OvEO. Figure 3a shows that the FTIR spectrum of alginate displays the expected characteristic signals, including O–H stretching at 3230 cm^−1^ and asymmetric and symmetric –COO^−^ stretches at 1593 and 1401 cm^−1^, indicating the ionic state of the carboxylic group. Additionally, C–O stretching can be observed at 1299 cm^−1^, C–O–C stretching of ether linkages in the glycosidic bonds of the polysaccharide appears at 1146 cm^−1^, and C–O stretching of sugar rings and ether linkages can be detected at 1022 cm^−1^ [29]. In the presence of Ca^2+^, the original signal at 1401 cm^−1^ of alginate alone shifts to 1411 cm^−1^ (Figure 3b), indicating the formation of crosslinks between the biopolymer and Ca^2+^, reinforcing the polymer network. Upon mixing alginate with Ca^2+^ and OvEO (Figure 3c), the O–H band shifts to 3193 cm^−1^, which is consistent with the formation of non-covalent hydrogen bond interactions between the phenolic compounds in the essential oil and alginate hydroxyl/ether oxygens. Furthermore, in this mixture, the signal at 1012 cm^−1^ suggests that the hydroxyl groups (–OH) of the phenolic compounds present in the OvEO establish hydrogen bonds with the oxygen atoms in the C–O linkages of the alginate, reducing the vibrational frequency and causing the observed shift [30]. Altogether, the data indicate physical encapsulation with non-covalent EO–alginate interactions.

### 3.2. Antifungal Activity of Hydrogel Beads Loaded with OvEO

The antifungal effect of OvEO-loaded hydrogel beads at different concentrations was evaluated against reference strains of *C. albicans*. Fluconazole was used as a control for the susceptible 90029 strain and for the resistant 10231 strain. The IC_50_ value was determined for each strain. For OvEO-loaded beads, we defined this value as the concentration of OvEO loaded in the beads capable of inhibiting the growth of the tested strains by 50%. Both strains were found to be susceptible to the treatment, exhibiting similar IC_50_ values. Although strain 90029 showed greater susceptibility, with a slightly lower IC_50_ value, this difference was not statistically significant (Table 2, *p* > 0.05).

**Figure 4:**
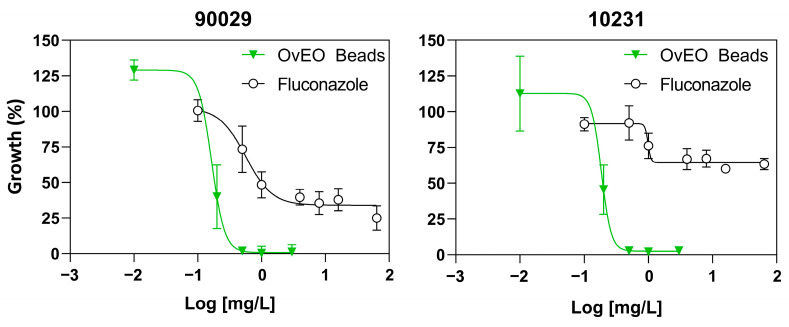
Growth inhibition of *C. albicans* by OvEO-loaded hydrogel beads. Dose–response curves were obtained from planktonic yeast cultures exposed to different concentrations of OvEO encapsulated in alginate hydrogel beads or fluconazole for 24 h at 37 °C. The curves for each strain represent the average of at least four independent experiments (n = 4).

Additionally, Figure 4 shows representative dose–response curves for each tested strain, showing that the OvEO-loaded hydrogel beads inhibited *C. albicans* growth in a dose-dependent manner in both strains.

### 3.3. Effect of OvEO-Loaded Hydrogel Beads on the Adhesion of C. albicans

The adhesion capacity of *C. albicans* is one of its key virulence factors, enabling this opportunistic pathogen to colonize host tissues, initiate biofilm formation, and increase its pathogenicity. For this reason, the effect of the fabricated hydrogel beads on the adhesion of *C. albicans* to an abiotic surface was evaluated. To define the concentrations of beads tested in this assay, the IC_50_ values obtained for each strain were used as references (0.5 × IC_50_, IC_50_, and 2 × IC_50_). The values are detailed as follows (Table 3).

**Figure 5:**
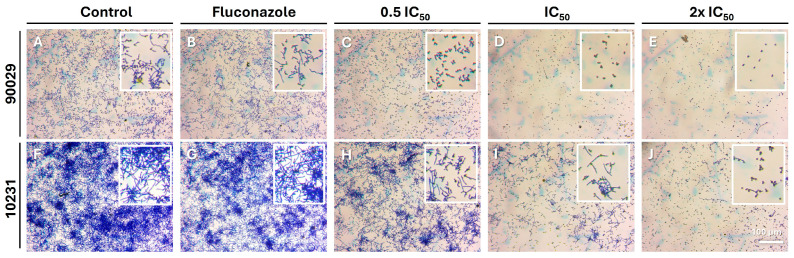
Effect of OvEO-loaded hydrogel on the adhesion of *C. albicans*. Representative images of adherent *C. albicans* cells from strains 90029 (**A**–**E**) and 10231 (**F**–**J**) stained with crystal violet in absence (control) or presence of treatments as indicated. IC_50_: concentration of OvEO present in the hydrogel beads that inhibited the growth of *C. albicans* strains by 50% (see Table 3 for details). Inserts show 10× magnification of representative morphological states of *C. albicans* for each condition.

Figure 5 displays representative images of the plate wells under the various experimental conditions of the adhesion assay. For strain 90029, the images show that both the untreated control and the sample treated with fluconazole exhibit a similar layer of adherent cells with a high presence of cells (Figure 5A,B). In the sample treated with hydrogel beads loaded with half the IC_50_, a less dense cell layer can be observed compared to the control (Figure 5C). Finally, the samples treated with hydrogel beads loaded with the IC_50_ and 2 × IC_50_ show a clear reduction in adherent cells, which is directly proportional to the concentration of OvEO present in the beads used. Strain 10231 formed a dense layer of cells with a large number of cell aggregates and a high presence of filamentous cells (Figure 5F). When the sample was treated with fluconazole, cell adhesion exhibited similar characteristics to the control (Figure 5G). In contrast, samples treated with hydrogel beads loaded with half the IC_50_ concentration showed a less dense cell layer with a noticeable reduction compared to the control, although filamentous cells were still highly present (Figure 5H). Finally, in samples treated with hydrogel beads loaded with the IC_50_ and 2 × IC_50_, cell density decreased even further, and the presence of filamentous cells was drastically reduced (Figure 5I,J).

**Figure 6:**
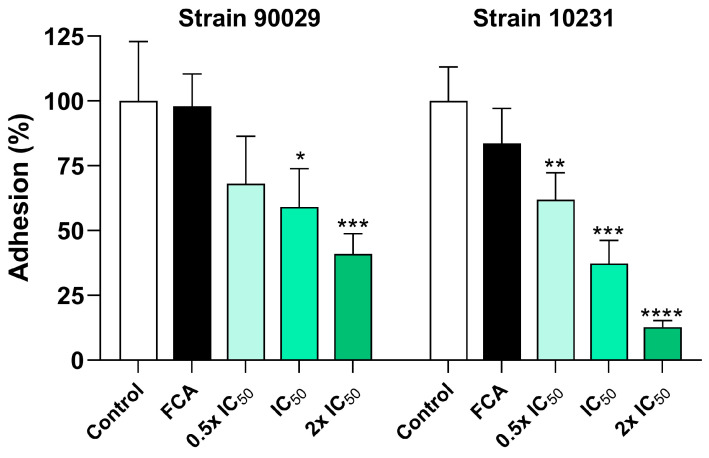
Inhibition of *C. albicans* adhesion by OvEO-loaded hydrogel beads at different concentrations. Bars represent the mean ± SD of the adhesion percentage from at least three independent experiments. * *p* < 0.05; ** *p* < 0.01; *** *p* < 0.001; **** *p* < 0.0001 compared to the control (one-way ANOVA with Tukey’s post hoc test). FCA: fluconazole (0.053 mg/mL); 0.5 × IC_50_: hydrogel bead loaded with half the IC_50_; IC_50_: hydrogel bead loaded with the IC_50_; 2 × IC_50_: hydrogel beads loaded with twice the IC_50_.

When quantifying the percentage of adhesion relative to the control, the results indicate that in the presence of fluconazole, the adhesion percentage did not decrease in a statistically significant manner in either of the tested strains. In contrast, for strain 10231, OvEO-loaded hydrogel beads produced a statistically significant reduction in the adhesion percentage at all tested concentrations compared to the control. A statistically significant difference was also observed between the effects of the hydrogel bead loaded with the lowest and the highest OvEO concentration (*p* = 0.0013). Regarding strain 90029, only the hydrogel beads loaded with the IC_50_ and twice the IC_50_ produced a statistically significant reduction in adhesion percentage compared to the control (Figure 6).

### 3.4. Determination of OvEO Release from the Hydrogel Beads

To monitor and quantify the release of OvEO from the fabricated hydrogel beads, it was necessary to examine the chemical composition of the OvEO and select a reference compound for measurement normalization. Thymol was chosen as the marker compound due to its solubility in aqueous media and its detectability in the UV–visible range. Initially, a spectral scan was performed using a thymol solution dissolved in RPMI-1640 medium (Sigma-Aldrich) at a known concentration, within the 200–400 nm wavelength range, to identify the peak absorbance value (272 nm) for use in subsequent measurements. In addition, a release measurement was conducted for the hydrogel beads loaded with the highest OvEO concentration (3%) (Supplementary Figure S1).

**Figure 7:**
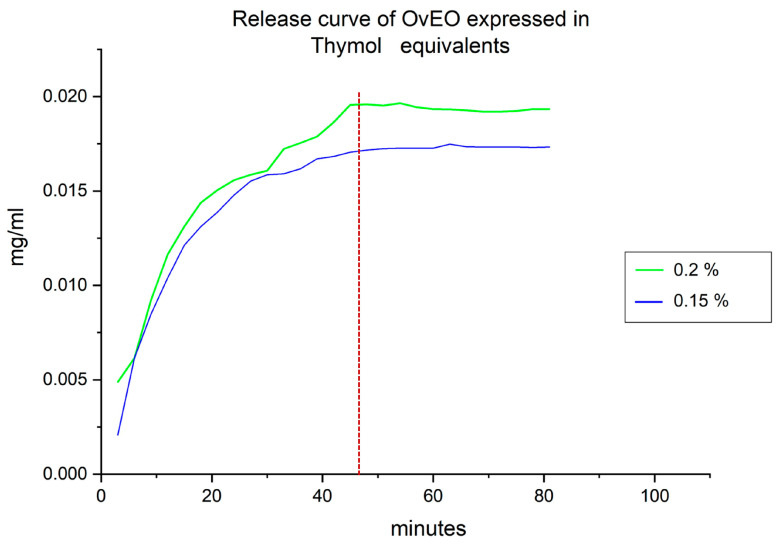
Release assay curve of OvEO expressed in thymol equivalents. The green curve represents the release profile of the beads prepared at the IC_50_ (0.2 mg/L) for strain 10231. The blue curve represents the release profile of the beads prepared at the IC_50_ (0.15 mg/L) for strain 90029. The red dashed line indicates the time when release becomes constant.

This information allowed us to use thymol as a marker of OvEO release from the beads. By using a calibration curve with a thymol standard, we obtained the following equation: Y = 26.009X + 0.053 (r = 0.99). Then, to construct the release curve as a function of time, the absorbance values obtained from the measurements of the beads were substituted into the linear equation derived from the thymol standard curve. In this way, the thymol concentration in mg/mL was determined, and these data were plotted as a function of time. For the hydrogel beads prepared at 0.15 mg/L (IC_50_ for the 90029 strain), the initially released concentration of thymol was 0.002 mg/mL, which progressively increased over time, reaching a maximum value of 0.017 mg/mL at 48 min. After reaching this peak, the thymol concentration stabilized, resulting in a release plateau. For the hydrogel beads prepared at 0.2 mg/L (IC_50_ for 10231 strain), the initial thymol concentration recorded was 0.005 mg/mL, which gradually increased until reaching a maximum value of 0.02 mg/mL at 48 min. After this point, the thymol concentration stabilized, and a release plateau was also achieved (Figure 7). In addition, after the construction of the release curves, a final measurement was performed on the disintegrated beads, yielding thymol concentrations of 0.017 mg/L and 0.02 mg/mL for the beads prepared at initial concentrations of 0.15 mg/L and 0.2 mg/L, respectively. These values were used for further calculations.

Finally, the quantification of the release percentage and the total amount of OvEO released from the hydrogel beads was carried out. To this end, the percentage of thymol release from the beads was initially quantified, as described in the Methods section. Release values of 100% were obtained for the beads prepared at both concentrations. Subsequently, the approximate amount of OvEO contained in the hydrogel beads was interpolated using the thymol standard curve. As a result, hydrogel beads prepared at 0.15 mg/L and 0.2 mg/L were found to contain 6.6 and 7.8 µg/mL of OvEO, respectively.

## 4. Discussion

The development of pharmaceutical formulations for the delivery of essential oils (EOs) offers several advantages, such as increased physical stability, protection against oxidation, reduced volatility, and controlled release. For this reason, numerous studies have focused on their development; however, those employing alginate hydrogels for this purpose are much more limited. In terms of the production of EO-loaded hydrogel beads, the results obtained in this study are consistent with previous reports that have described the formation of stable, spherical hydrogel beads loaded with essential oil, exhibiting uniform size and an average diameter of approximately 2.1 ± 0.1 mm [24]. Additionally, the findings observed in the SEM images of the OvEO-loaded hydrogel beads are in agreement with previous studies, which also reported folding and stacking of the polymer matrix, along with the formation of porous surfaces. These morphological alterations are attributed to water trapped between alginate polymers prior to freeze-drying, as well as to the freeze-drying process itself, which can compromise the structural integrity of the alginate beads [24,31]. Moreover, a recurrent observation is the presence of crystals within the structure of the alginate beads in the SEM images. However, further comparison regarding their chemical composition is not possible, as those studies did not perform EDX analysis and only hypothesized that the crystals might correspond to sodium chloride (NaCl) distributed on the surface, resulting from ion exchange and its subsequent release into the hardening medium during bead formation.

The available evidence on the incorporation of EOs into vehicles formed by biopolymeric frameworks (hydrogels) used in the treatment of *C. albicans* infections reports antifungal and antibiofilm effects, which is consistent with the results obtained in this study. However, the methodology employed to define *C. albicans* susceptibility to the treatment is mostly qualitative, using disc diffusion assays in most cases [32,33,34]. In this study, our approach was quantitative, enabling us to adapt the methodology for antifungal drugs in the context of hydrogel bead vehicles loaded with OvEO, thus establishing a precedent for reference methods to assess *C. albicans* susceptibility to EOs incorporated into these types of pharmaceutical formulations. Considering this, we can compare the results obtained with those previously reported in a study by Cid-Chevecich et al. (2022), where the IC_50_ values obtained for strains 90029 and 10231 were 0.01 mg/L and 0.97 mg/L, respectively, and the growth inhibition curve exhibited dose-dependent behavior [15]. In our study, the IC_50_ values for *C. albicans* strains 90029 and 10231 were 0.15 mg/L and 0.2 mg/L, respectively, and the growth inhibition curve was also dose-dependent. The difference in the values reported in both studies may be explained by the fact that in the study by Cid-Chevecich et al. (2022) [15], the OvEO was added directly to the yeast and biofilms, while in our case, the release profile could affect the result. On the other hand, both formulations inhibited *C. albicans* growth in a dose-dependent manner.

Regarding the effectiveness of OvEO-loaded hydrogel beads on *C. albicans* adhesion, the results obtained in this study can be compared with those reported by Cid-Chevecich et al. (2022) [15], who evaluated the effect of OvEO in an adhesion assay on abiotic surfaces. The authors reported that OvEO inhibited adhesion by approximately 60% in both strains (90029 and 10231). This trend is clearly reflected in the images of the adhered cells, which also revealed that, in strain 90029, samples treated with OvEO-loaded hydrogel beads predominantly exhibited yeast morphology. In contrast, the control group and the fluconazole-treated samples showed mostly filamentous cells (hyphae/pseudohyphae). For strain 10231, the predominant morphology of the adhered cells progressively changed with increasing concentrations of OvEO-loaded beads. Specifically, in the sample treated with the lowest concentration, most of the observed cells displayed a filamentous morphology, whereas in the sample treated with the highest concentration, the majority of the cells exhibited yeast forms. As the concentration of the beads increased, the prevalence of filamentous cells decreased consistently in both strains. This phenomenon could be associated with previous findings reported by Cid-Chevecich et al. (2022) [15], who determined that OvEO inhibited filamentation by 35% and 41% in strains 90029 and 10231, respectively, an effect with direct implications for biofilm formation capacity. Nonetheless, to confirm this hypothesis, assays specifically aimed at evaluating morphogenesis changes are required to determine the effect of OvEO-loaded hydrogel beads on filamentation, as well as to assess their influence on biofilm formation ability.

Finally, concerning the controlled release capacity of EO from the hydrogel beads, the data obtained can be compared with the study conducted by Gholamian et al. (2021) [24], in which calcium alginate hydrogel beads filled with cumin seed essential oil (CsEO) were fabricated by injecting drops of an essential oil–sodium alginate emulsion into a CaCl_2_ hardening solution. In that study, the release of CsEO from the hydrogel beads was evaluated in simulated gastrointestinal fluids, with a reported release time of 180 min. In contrast, the release time obtained in our study was 48 min. This difference suggests that the fabrication methodology proposed in that study may offer advantages over the one employed in our work. Therefore, if an optimization aimed at prolonging the OvEO release time from our hydrogel beads is required, this would be a key aspect to consider in future research following our line of investigation. Similarly, the percentage of EO released from hydrogel beads can also be analyzed. The results reported by Gholamian et al. (2021) [24] indicate a release percentage of 96%, and the authors concluded that no chemical interaction occurs between CsEO and the alginate hydrogel. These findings are consistent with those obtained in the present study, where a release percentage of 100% was reached. Additionally, FTIR analysis confirmed that, like CsEO, OvEO does not form chemical bonds with the alginate hydrogel, which explains the high release percentages observed in both cases.

Although our results are promising, some limitations should be addressed in future studies. The release assay quantified the essential oil (OvEO) using thymol (its predominant water-soluble constituent) as a single marker. As a result, the method primarily captures the kinetics of the hydrophilic fraction and may underrepresent non-polar or highly volatile terpenoids. While low-cost and amenable to rapid time-course measurements, this approach should be complemented by analyses of additional constituents, particularly non-polar terpenoids. Another limitation is that the antifungal and antibiofilm activity of the OvEO-containing hydrogels was evaluated only against reference strains of *C. albicans*; testing against clinical isolates, which often exhibit distinct growth kinetics, susceptibility profiles, and biofilm-forming capacities, is warranted. In addition, toxicological studies are needed to elucidate the effects of the oil-loaded beads on mammalian cells, providing data to evaluate performance in more complex models and to support future clinical translation. Finally, successful scale-up of OvEO-loaded hydrogel beads will require optimization of parameters not addressed here, including polymer concentration (to adjust release kinetics), crosslinking conditions, and bead size, all of which can influence mechanical properties and biological activity.

In summary, our results demonstrate that OvEO-loaded hydrogel beads exhibit significant antifungal effects on *C. albicans*, suggesting their potential as a promising antifungal strategy. These findings align with previous research and provide new insights into the development of bioactive materials aimed at preventing fungal colonization. Future studies should focus on in vivo evaluations and the long-term stability of these systems to further validate their clinical applicability.

## 5. Conclusions

The development of pharmaceutical formulations for the delivery of EOs with biological activity against *C. albicans* is a rapidly growing area of research. The results obtained in this study support the potential of alginate-based hydrogels as a viable delivery system for this purpose. The OvEO-loaded hydrogel beads formulated in this study proved to be a stable formulation with effective encapsulation capacity, enabling the gradual release of OvEO. FTIR analysis, complemented by SEM imaging, confirmed the successful encapsulation of OvEO within the hydrogel matrix, without forming chemical bonds between the components. Moreover, the OvEO-loaded hydrogel beads exhibited dose-dependent antifungal activity. Notably, the hydrogels loaded with OvEO effectively inhibited *C. albicans* adhesion to abiotic surfaces by 60–80%. Given the straightforward production process and the relatively low manufacturing costs, these findings encourage further research into the in vivo efficacy and biocompatibility of OvEO-loaded hydrogels to advance their clinical use in the prevention and treatment of *C. albicans* infections.

## Figures and Tables

**Table 1:** Formulation of O/W emulsions containing OvEO in sodium alginate hydrogel.

Sodium Alginate Hydrogel (mL)	OvEO (µL)	Essential Oil Concentration (*v*/*v*)
7 mL	0 µL	0%
7 mL	0.7 µL	0.01%
7 mL	14 µL	0.2%
7 mL	35 µL	0.5%
7 mL	70 µL	1.0%
7 mL	210 µL	3.0%

**Table 2:** Concentration of OvEO loaded in the beads capable of inhibiting 50% of the growth (IC_50_) against *C. albicans* strains.

*C. albicans* Strain	90029	10231	*p* Value (*t*-Test)
**OvEO** **(mg/L)**	0.15 ± 0.05	0.2 ± 0.06	0.4954
**Fluconazole** **(mg/L)**	0.53 ± 0.09	>64 mg/L	

The results are expressed as the mean ± SD of three independent experiments (n = 3). The IC_50_ values obtained for each strain were used in subsequent assays.

**Table 3:** Concentration of OvEO-loaded hydrogel beads used in the adhesion assay.

Strain	0.5 × IC_50_	IC_50_	2 × IC_50_
**90029**	0.075 mg/L	0.15 mg/L	0.30 mg/L
**10231**	0.10 mg/L	0.20 mg/L	0.40 mg/L

## Data Availability

The original contributions presented in this study are included in the article/Supplementary Material. Further inquiries can be directed to the corresponding author.

## Supplementary Materials

The following supporting information can be downloaded at: https://www.mdpi.com/article/10.3390/microorganisms13092065/s1, Supplementary Figure S1. UV absorbance spectrum scan for thymol stock solution (0.1 mg/mL).

## Abbreviations

The following abbreviations are used in this manuscript:

EO	Essential oil
OvEO	*Origanum vulgare* essential oil
IC_50_	Inhibitory concentration 50
