# Supplementary material for: Characterization of Hydrogel Beads for the Gradual Release of Origanum vulgare L. Essential Oil and Evaluation of Their Antifungal Activity Against Candida albicans
Source: Microorganisms. 2025 Sep 5;13(9):2065. doi: 10.3390/microorganisms13092065 (PMC12472747; doi:10.3390/microorganisms13092065)
Supplement: Supplementary file 1 [file microorganisms-13-02065-s001.zip › microorganisms-3820224-supplementary.pdf]

Supplementary Materials

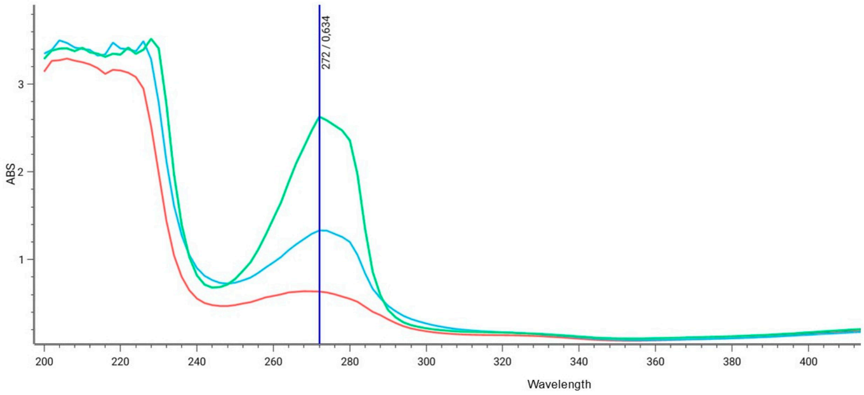

Supplementary Figure S1. UV absorbance spectrum scan for thymol stock solution (0.1 mg/mL). Absorbance (ABS) was plotted as a function of wavelength. The green curve indicates the absorbance peak of thymol at 272 nm, the light blue curve represents the absorbance signal of hydrogel beads loaded with OvEO (3%), and the red curve corresponds to the absorbance of the RPMI-1640 medium (Sigma-Aldrich).
